# Immune regulatory gene polymorphisms, frequent cannabis use, and psychosis: implications to Treg hypofunction

**DOI:** 10.1192/j.eurpsy.2022.1798

**Published:** 2022-09-01

**Authors:** F. Corsi-Zuelli, C. Loureiro, R. Shuhama, D. Quattrone, B. Deakin, P. Menezes, R. Lacchini, F. Coeli-Lacchini, P. Louzada-Junior, C. Del-Ben

**Affiliations:** 1 University of São Paulo, Ribeirão Preto Medical School, Neuroscience And Behaviour, Ribeirão Preto, Brazil; 2 King’s College London, Social, Genetic And Developmental Psychiatry Centre, Institute Of Psychiatry, Psychology And Neuroscience, London, United Kingdom; 3 University of Manchester, Division Of Neuroscience And Experimental Psychology, Manchester, United Kingdom; 4 University of São Paulo, Faculty of Medicine, Department Of Preventive Medicine, São Paulo, Brazil; 5 University of São Paulo, Ribeirão Preto College of Nursing, Brazil, Psychiatric Nursing, Ribeirão Preto, Brazil; 6 University of São Paulo, School of Pharmaceutical Sciences, Brazil, Department Of Clinical Analyses, Toxicology And Food Science, Ribeirão Preto, Brazil; 7 University of São Paulo, Ribeirão Preto Medical School, Brazil, Internal Medicine, Ribeirão Preto, Brazil

**Keywords:** Psychosis, Adenosine, Cannabis, Immune system

## Abstract

**Introduction:**

We have previously shown that the association between frequent cannabis use and psychosis is more likely in subgroups with low-grade inflammation than subgroups without (PMID: 33736715). The role of immune-related polymorphisms remains unknown.

**Objectives:**

To explore whether polymorphisms affecting the function of key immune regulatory proteins moderate the association between cannabis and psychosis, namely: *ENTPD1* and *NT5E*, involved in the synthesis of CD39, CD73, respectively, and anti-inflammatory adenosine; *CTLA4* and *FOXP1,* essential for Treg functional capacity.

**Methods:**

We genotyped blood samples from 283 community-based controls and 140 recent-onset psychosis patients in Brazil (EU-GEI consortium, Ribeirão Preto/SP) for twelve polymorphisms (*ENTPD1*: rs3814159, rs3176891, rs10748643; *NT5E:* rs9444348, rs2295890; *CTLA4*: rs3087243, rs231775, rs5742909, rs4553808; *FOXP1*: rs6803008, rs6786408, rs830599; Illumina Human Core Exome-24). Cannabis frequency (daily, less than daily, never) was assessed by self-report (Cannabis Experience Questionnaire). Binary logistic regression models (OR,95%CI) included case status as the outcome, genotype (dominant model), cannabis frequency, and an interaction term between the two as exposure, adjusting for confounders (age, sex, ethnicity, tobacco smoking).

**Results:**

We found significant interactions between cannabis use and polymorphisms for *ENTPD1* (rs3814159), *NT5E* (rs9444348), and *FOXP1* (rs6786408). Less than daily or daily use were, in a dose-response fashion, only associated with psychosis in those with the variant and heterozygous genotypes; less than daily: *ENTPD1* AG/GG (3.34,1.71-6.50); *NT5E* AG/AA (3.71,1.87-7.33); *FOXP1* AC/CC (2.98,1.54-5.77); daily: *ENTPD1* AG/GG (16.81;5.89-47.96); *NT5E* AG/AA (21.20,6.81-66.01); *FOXP1* AC/CC (13.75,5.22-36.21).

**Conclusions:**

Variation in genes that affect Treg function appears to modify the effect of cannabis consumption on psychosis in keeping with Treg hypofunction hypothesis (PMID:33713699).

**Disclosure:**

No significant relationships.

